# Reducing the acquisition time for magnetic resonance imaging using super-resolution image generation and evaluating the accuracy of hippocampal volumes for diagnosing Alzheimer’s disease

**DOI:** 10.3389/fneur.2025.1507722

**Published:** 2025-07-15

**Authors:** Nobukiyo Yoshida, Hajime Kageyama, Hiroyuki Akai, Satoshi Kasai, Kei Sasaki, Noriko Sakurai, Naoki Kodama

**Affiliations:** ^1^Faculty of Medical Technology, Department of Radiological Technology, Niigata University of Health and Welfare, Niigata, Japan; ^2^Department of Radiology, Institute of Medical Science, The University of Tokyo, Tokyo, Japan; ^3^Graduate Division of Health Sciences, Komazawa University, Tokyo, Japan; ^4^Department of Intelligent Information Engineering, Research Promotion Unit, School of Medical Sciences, Fujita Health University, Toyoake, Japan; ^5^Department of Radiological Sciences, Faculty of Medical Science and Technology, Gunma Paz University, Gunma, Japan

**Keywords:** Alzheimer disease, Pix2Pix network, super-resolution, hippocampal volume measurement, brain anatomical analysis using diffeomorphic deformation

## Abstract

**Introduction:**

Brain magnetic resonance imaging (MRI) is important for diagnosing Alzheimer’s disease (AD), and MRI acquisition time should be reduced. The current study aimed to identify which Pix2Pix-based super-resolution images can reduce errors associated with brain anatomical analysis with diffeomorphic deformation examination and MRI acquisition time.

**Methods:**

Fifty patients with dementia who uderwent scanning using a 3-T MRI scanner in the OASIS-3 database were used to construct a super-resolution network. Network training was performed using a scaled image (64 × 64) down-sampled from the original image as the input image and paired with the original high-resolution (256 × 256) supervised image. The hippocampal volume was measured using brain anatomical analysis with diffeomorphic deformation software, which employs machine learning algorithms and performs voxel-based morphometry. Peak signal-to-noise ratio (PSNR) and Multiscale structural similarity (MS-SSIM) score were used to objectively evaluate the generated images.

**Results:**

At *λ* = e^3^, the PSNR and MS-SSIM score of the generated images were 27.91 ± 1.78 dB and 0.96 ± 0.0045, respectively. This finding indicated that the generated images had the highest objective evaluation. Using the images generated at *λ* = e^4^, the left and right hippocampal volumes did not significantly differ between the original and generated super-resolution images (*p* = 0.76, *p* = 0.19, respectively).

**Discussion:**

With super-resolution using the Pix2Pix network, the hippocampal volume can be accurately measured, and the MRI acquisition time can be reduced. The proposed method does not require special hardware and can be applied to previous images.

## 1 Introduction

Alzheimer’s disease (AD) had already affected 500,000 people in 2020. The incidence of AD can increase by 60% from 2020 to 2030 and by >200% by 2050 (1). AD is diagnosed using different diagnostic methods including cognitive testing, physical examination, and spinal fluid and blood analysis (2, 3). Brain magnetic resonance imaging (MRI) facilitates a noninvasive structural assessment and plays an important role in AD diagnosis (4). Patients with AD exhibit extensive and progressive synaptic and neuronal loss. Therefore, for the early diagnosis of AD, neuroimaging is essential to examine the pattern of changes in the preclinical stages of the disease (5).

Voxel-based morphometry (VBM) analysis is performed to calculate the gray and white matter volume and cerebrospinal fluid in voxel units by transforming and spatially normalizing MRI data based on standard brain coordinates (6). The hippocampal volume can be measured with high accuracy using the brain anatomical analysis with the diffeomorphic deformation (BAAD) software, which conducts VBM analysis and uses machine learning algorithms for diagnosing AD and predicting the progression of mild cognitive impairment (7). To measure hippocampal volume using the software, three-dimensional T1-weighted imaging (T1WI) MRI with a thin slice thickness of 1.0–1.5 mm is required to include the whole head without gaps in the imaging range. However, three-dimensional T1WI has a long scanning time (8). Compressed sensitivity encoding (SENSE) is a method that combines the SENSE method and compressed sensing technology for reducing MRI acquisition time. It was developed to achieve a higher speed and image quality (9). Another technique, controlled aliasing in parallel imaging results in a higher acceleration technology, which modifies the algorithm of the image acquisition method (k-space), reduces imaging time, and achieves a high spatial resolution with a thin slice thickness (10). Nevertheless, these scanning techniques require hardware or software modifications that involve significant investment.

The applications of deep learning techniques in medical imaging are promising. Goodfellow et al. proposed a generative adversarial network (GAN) (11). In the field of super-resolution technology, Ledig et al. implemented a GAN for image super-resolution tasks, which outperformed previous algorithms in terms of image perceptual quality metrics (12). Pix2Pix, a type of GAN, was reported as an image-specific network that transforms one image into another by learning pairs of images (13). Previous studies have reported the applications of MRI using Pix2Pix. For example, it suppresses head motion artifacts, improves fat-suppression methods in mammograms, and provides super-resolution techniques for magnetic resonance angiography images (14–16).

MRI acquisition time is closely related to the number of phase-encoding steps. Reducing these steps shortens the scan time but often results in compromised spatial resolution and image quality. Although various acquisition techniques have been developed to mitigate this trade-off, maintaining sufficient image quality remains challenging in clinical scenarios involving patient motion, limited cooperation, or strict time constraints (17).

Artificial intelligence based super-resolution techniques offer a complementary approach by enhancing image quality through post-processing rather than acquisition adjustments. Recent studies have shown that artificial intelligence based super-resolution can reduce MRI acquisition time by up to 45% without degrading diagnostic quality (18). However, whether super-resolution images reconstructed from low-resolution inputs can be reliably used for quantitative brain analysis, particularly hippocampal volumetry, using BAAD software remains unclear.

We hypothesized that high-resolution brain MRI images suitable for quantitative analysis could be reconstructed from low-resolution inputs using a Pix2Pix network, which is a type of GAN. This study aimed to evaluate whether super-resolution images generated by GAN based approach can preserve the accuracy required for BAAD analysis. In addition, this study investigated whether tuning the *λ* hyperparameter in the Pix2Pix network contributes to the generation of super-resolution images appropriate for accurate volumetric assessment under limited imaging conditions.

## 2 Materials and methods

### 2.1 Participants and image acquisition

The Institutional Committee of Niigata University of Health and Welfare approved this study (approval no. 19097–230,718). A written informed consent was not obtained because the study used the MRI data of patients with AD in the OASIS-3 database (https://sites.wustl.edu/oasisbrains/).

### 2.2 Selection criteria

#### 2.2.1 Selection criteria for the training dataset

To construct a model that considers the complex patterns of brain atrophy in AD, the training dataset included 50 patients diagnosed with AD, with a clinical dementia rating (CDR) score of ≥0.5, and consecutive patients registered in the OASIS-3 database (OAS-ID: 111–1,140) who have imaging data obtained within 1 year before or after the date of diagnosis. Further, using a 3-T MRI scanner, the scanning conditions were based on T1WI with a matrix size of 176 × 256, pixel size of 1 mm, and 256 slices. The age of the participants was 74.98 ± 8.55 years, the CDR was 0.83 ± 0.34, and the male-to-female ratio was 25:25.

#### 2.2.2 Selection criteria for the testing dataset

The testing dataset comprised 20 consecutive participants who were identified using their registration ID (OAS-ID: 1–37) in the OASIS-3 database. For the testing dataset, an objective evaluation of super-resolution images was performed to evaluate the presence of brain atrophy. Therefore, the diagnostic results and CDR scores of the participants were excluded from the selection criteria. The MRI conditions of the testing dataset were similar to those of the training dataset. The age of the participants was 68.74 ± 8.67 years, the CDR was 0.13 ± 0.28, and the male-to-female ratio was 7:13. Table 1 shows the training and testing datasets.

**Table 1:** Details of the training and testing datasets.

Parameter	Training dataset		Testing dataset	
Number	50		20	
Tesla	3		3	
Age Mean ± SD	74.98 ± 8.55		68.74 ± 8.67	
CDR	0.83 ± 0.34		0.13 ± 0.28	
Sex	M:25	F:25	M:7	F:13

SD, standard deviation; CDR, clinical dementia rating; M, male; F, female.

### 2.3 Super-resolution (Pix2Pix) network

#### 2.3.1 Dataset and pre-prosessing

The Pix2Pix network was constructed to generate super-resolution images. The network was based on the official TensorFlow tutorial (https://www.tensorflow.org/tutorials/generative/pix2pix?hl=ja) and adapted to MRI input. The GeForce GTX 2080Ti graphics card (Nvidia Corporation, Santa Clara, CA, the USA) with a base clock speed of 2.1 GHz/s (corei7-13700), memory bandwidth of 64 GB/s, and memory per board of 11 GB was used. Further, the following software were utilized: Python 3.7.16 (Python Software Foundation, Delaware, the USA), TensorFlow-GPU 2.10.0, Spyder 4.1.3, and Keras 2.3.1 (Google, Mountain View, Calif, the USA).

The training T1WI axial images (176 × 256) acquired from a OASIS-3 public database were zero-filled with 40 pixels on each side, extending the matrix size to 256 × 256. This zero-filling was applied because the GAN generator architecture, U-Net, is fundamentally designed to accept square input images (19). Although U-Net can process rectangular images, maintaining input–output size consistency requires modifications such as changing convolution strides or kernel shapes, which can lead to uneven processing accuracy along different dimensions (20). Therefore, zero- filling was used to avoid altering the original image content while ensuring consistent and stable network performance.

The zero-filled areas do not contribute to spatial resolution improvement and were only added to meet network input requirements. These zero-filling regions were removed during post-processing to prevent artificially inflated image similarity metrics such as PSNR and MS-SSIM. Hence, zero-filling was not intended to increase the acceleration factor or enhance true spatial resolution. These images were down-sampled (64 × 64) using bicubic interpolation to obtain input images. The original zero-filled T1WI images (256 × 256) were used as the supervised datasets. In addition, 56 images of the parietal side were excluded from the training dataset due to the presence of several signal-free region effects. In this study, 200 consecutive images of the foot side were collected from 50 patients, with a total of 10,000 paired images trained on the Pix2Pix network.

**Figure 1:**
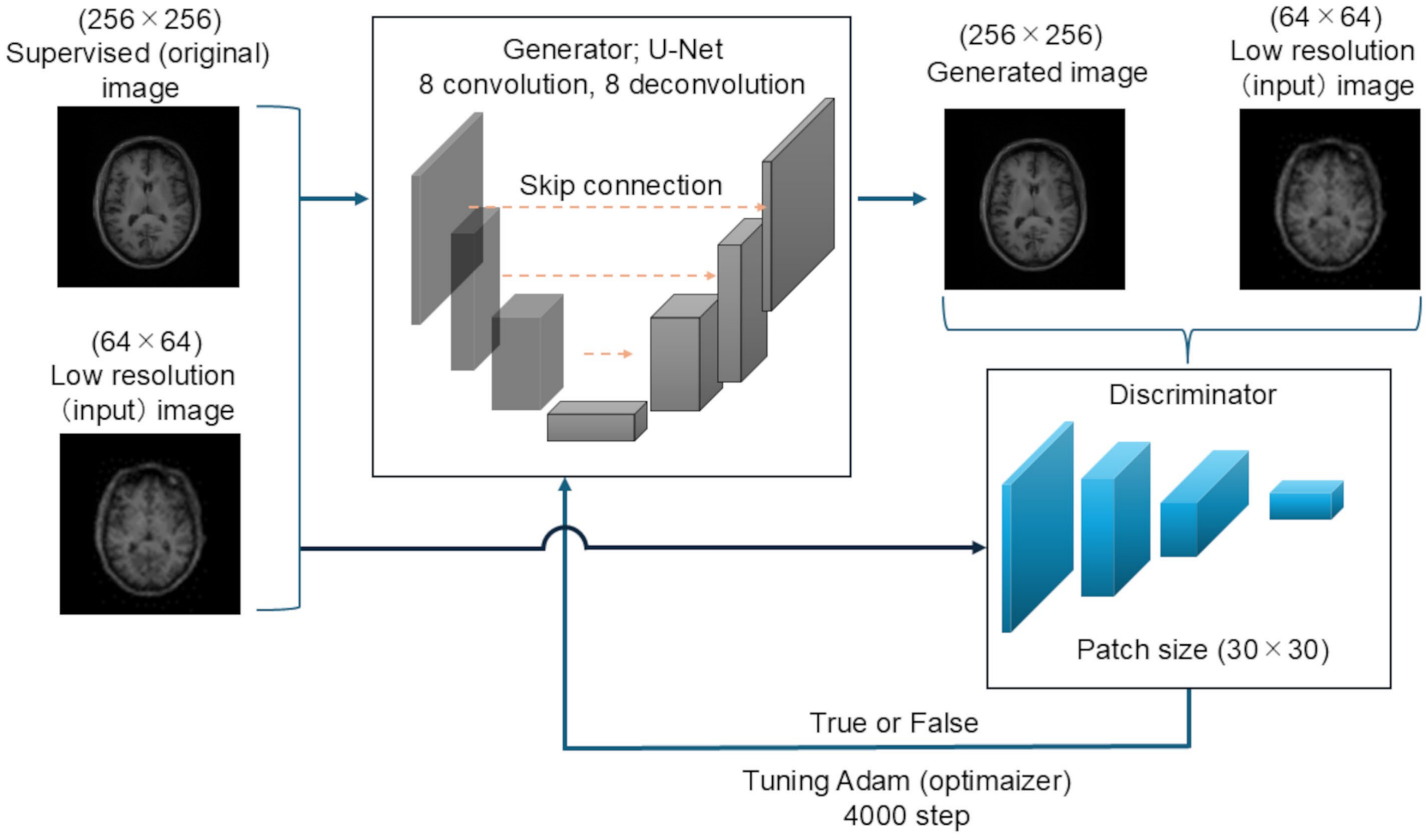
Schematic of the super-resolution network. U-Net was used as the generator network. This network comprised an encoder with eight convolution layers and a decoder with the same number of deconvolution layers. Skip connections were performed. PachGAN was utilized as the discriminator network, and the patch size was set to 30 × 30. The Adam optimizer was applied as the learning parameter, and 4,000 learning steps were performed.

The testing images were first zero-filled in the same manner as the training images, and the matrix size was changed to 256 × 256. Next, the images were down-sampled, and the matrix size was changed to 64 × 64. The testing images were generated using the 256 images of each patient registered in the OASIS-3 dataset. Figure 1 shows the schematic of the Pix2Pix network used in this study.

### 2.4 Generator

The generator network was based on U-Net (19), with a skip connection comprising an encoder block and decoder block. The encoder block contained eight convolution layers, and convolution was performed in each block prior to batch normalization and activation using a leaky rectified linear unit. The decoder block comprised the same number of up-sampling layers. In the encoder stack, inverse convolution, batch normalization, dropout (applied to the first three blocks), and activation with the rectified linear unit function were applied in that order. Then, batch normalization was applied to the output.

### 2.5 Discriminator

PatchGAN was used as the discriminative network. PatchGAN divides the image into small regions (patches) of a specific size and compares each patch for binary classification, rather than directly comparing the whole image. In this study, the patch size was set to 30 × 30. First, the original and generated images of each patch were convolved once. Second, the output of each patch was concatenated, further convolved, and converted to one dimension for complete combination. Finally, all patches were concatenated for final binary (true-false) classification using the softmax function.

The Adam optimizer was used as the learning parameter, with an initial learning rate of 2e-4 and β1 of 0.5, and 4,000 steps were used (21). The training time of the proposed model was 8 h and 30 min.


$$G*=\arg\;\min_{G}.\underset{D}{\;\max}\;\;L_{c}.\mathrm{GAN}(G,D)+\lambda .L_{L1}\;(G)$$

The Pix2Pix loss function was calculated using the following equation:

According to a current research on super-resolution magnetic resonance angiography images using Pix2Pix as a reference (16), experiments were conducted by setting the value of λ, which has a significant contribution to the loss of the generator network, to seven different values: e^0^, e^1^, e^2^, e^3^, 5 × e^3^, e^4^, e^5^.

### 2.6 Postprocessing

These zero-filled regions were subsequently removed during postprocessing to ensure that evaluation metrics such as PSNR and MS-SSIM accurately reflect the reconstruction quality of the original image content, rather than being artificially inflated by the zero-filled areas. The digital imaging and communications in medicine (DICOM) information required for the VBM analysis was set identical to the corresponding original images. The corresponding DICOM tags were added to the generated and super-resolution images using the MATLAB software R2023b (Mathworks, Natick, Massachusetts, the USA). The input image, a down-sampled image (64 × 64), was up-sampled to 256 × 256 using the nearest neighbor algorithm, and VBM analysis was performed with the same DICOM information as the corresponding original image.

### 2.7 Objective evaluation

The images were not generated well when set at *λ* = e^0^ and e^1^. Thus, an objective evaluation when set at λ = e^2^ to e^5^ was performed. The PSNR and MS-SSIM of the generated super-resolution and original images were calculated using the MATLAB software. Two objective evaluations were performed for each pair of supervised and generated images. The average for each patient was used as the basis of the study. The BAAD software package (version 4.3.2.0) was utilized to measure the left and right hippocampal volume.

### 2.8 Statistical analysis

The differences in the average PSNR and MS-SSIM scores between the five types of images (*λ* = e^2^, e^3^, 5 × e^3^, e^4^, e^5^) and the original images were assessed using the Friedman test, treating each *λ*–original image pair as a repeated measure. When the Friedman test indicated a significant difference, *post hoc* pairwise comparisons between the generated and original images at each λ were conducted using the Wilcoxon signed-rank test. Additionally, the left and right hippocampal volumes obtained from the original and generated images were compared separately for each λ using the Wilcoxon signed-rank test. The significance level in the Friedman test was set at *p* < 0.005 using Bonferroni correction for the number of comparisons. The significance level in the Wilcoxon signed-rank test was set at *p* < 0.05. Statistical analyses were performed using the EZR software (22).

## 3 Results

### 3.1 Generated images

**Figure 2:**
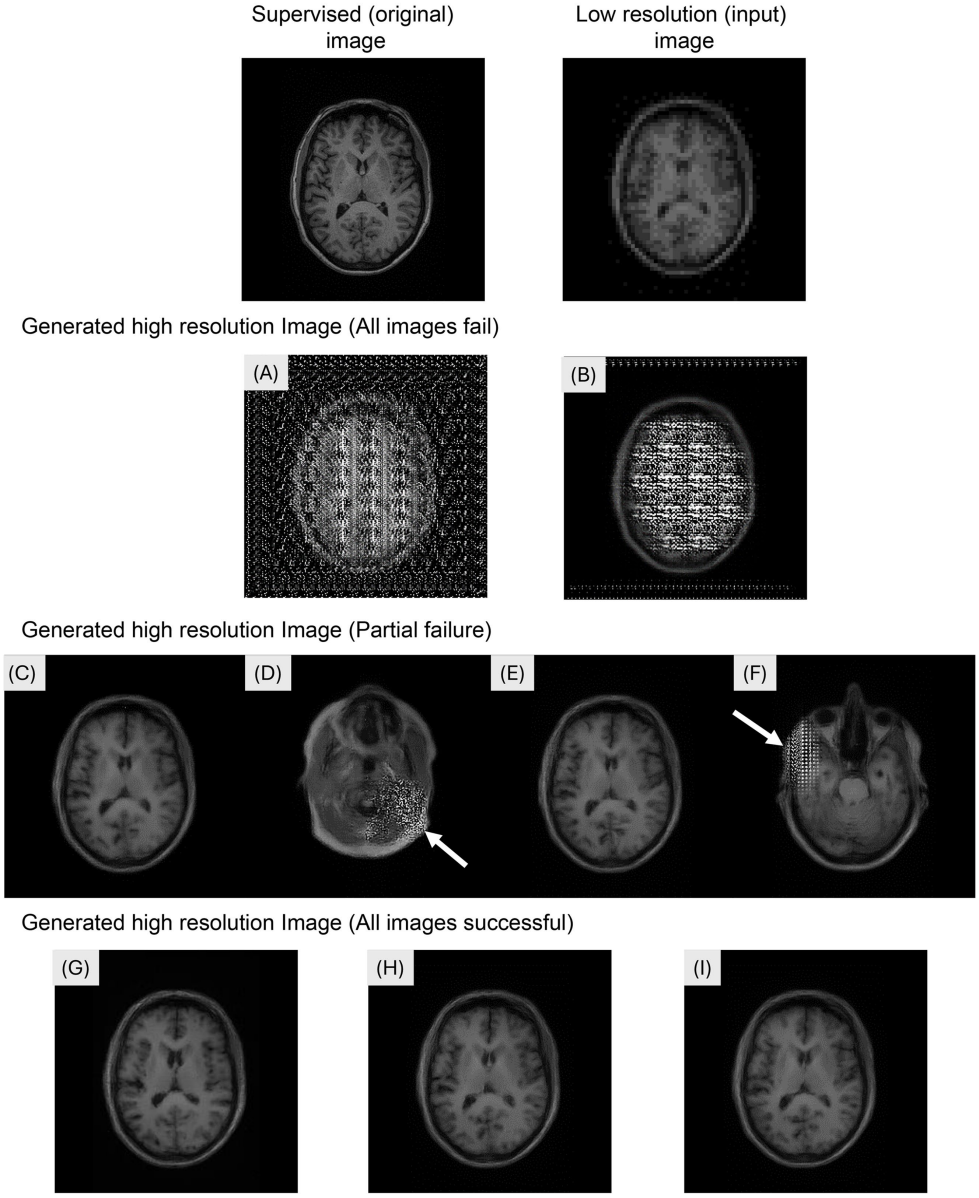
Examples of the input, supervised, and generated images in the Pix2Pix network. The images were generated by modifying the hyperparameter lambda. **(A)** *λ* = e^0^, **(B)** λ = e^1^: Mosaic-like noise was observed throughout the brain. **(C,D)** λ = e^2^: Super-resolution images were successfully generated; however, mosaic-like noise was observed in 29.5% of slices (1,509/5,120 slices). **(E,F)** λ = e^3^: Super-resolution images were generated with mosaic-like noise observed in 24.9% of slices (1,277/5,120 slices). **(G)** λ = 5 × e^3^, **(H)** λ = e^4^: **(I)** λ = e^5^: Super-resolution images were successfully generated for all images.

The generation of super-resolution images from 5,120 (256 × 20 participants) input images took 126 s. Figure 2 shows the examples of the input, supervised, and generated images in the Pix2Pix network. Mosaic-like noise was observed throughout the brain on the images generated at *λ* = e^0^ (Figure 2A) and *λ* = e^1^ (Figure 2B). Super-resolution images were successfully generated at *λ* = e^2^ (Figure 2C) and λ = e^3^ (Figure 2E). Super-resolution images were successfully generated at λ = e^2^ (Figure 2C) and *λ* = e^3^ (Figure 2E). However, visual inspection revealed that mosaic-like noise was observed in 29.5% of slices (1,509/5,120 slices) at *λ* = e^2^ (Figure 2D) and in 24.9% of slices (1,277/5,120 slices) at *λ* = e^3^ (Figure 2F). Mosaic-like noise was not observed on images generated at *λ* = 5 × e^3^ (Figure 2G), at λ = e^4^ (Figure 2H) and *λ* = e^5^ (Figure 2I), and super-resolution was successful in all images.

### 3.2 Objective evaluation

Table 2 shows the PSNR and MS-SSIM scores. The PSNRs (mean ± standard deviation [SD]) of the generated images relative to the original images were 27.14 ± 2.30 dB at λ = e^2^, 27.91 ± 1.78 dB at λ = e^3^, 27.10 ± 1.69 dB at λ = 5 × e^3^, 27.65 ± 1.78 dB at λ = e^4^, and 26.94 ± 2.28 dB at λ = e^5^. The MS-SSIM scores (mean ± SD) were 0.95 ± 0.0073 at λ = e^2^, 0.96 ± 0.0045 at λ = e^3^, 0.95 ± 0.0048 at *λ* = 5 × e^3^, 0.96 ± 0.0047 at *λ* = e^4^, and 0.95 ± 0.0057 at *λ* = e^5^. The PSNR and MS-SSIM scores of the images generated at *λ* = e^2^, …, e^5^ significantly differed (*p* < 0.005) based on the Friedman test. Further, the PSNR and MS-SSIM scores of the images generated at *λ* = e^3^ significantly increased, with the highest value being e^2^ < e^3^, 5 × e^3^ < e^3^, e^4^ < e^3^, and e^5^ < e^3^ (Wilcoxon signed-rank test, *p* < 0.05).

**Table 2:** PSNR and MS-SSIM scores.

λ	e^2^	e^3^	5 × e^3^	e^4^	e^5^
PSNR [dB]	27.14 ± 2.3	27.91 ± 1.78	27.10 ± 1.69	27.65 ± 1.78	26.94 ± 2.28
MS-SSIM	0.95 ± 0.0073	0.96 ± 0.0045	0.95 ± 0.0048	0.96 ± 0.0047	0.95 ± 0.0057

PSNR, peak signal-to-noise ratio; MS-SSIM, Multi-Scale Structural Similarity.

### 3.3 Hippocampal volume

**Figure 3:**
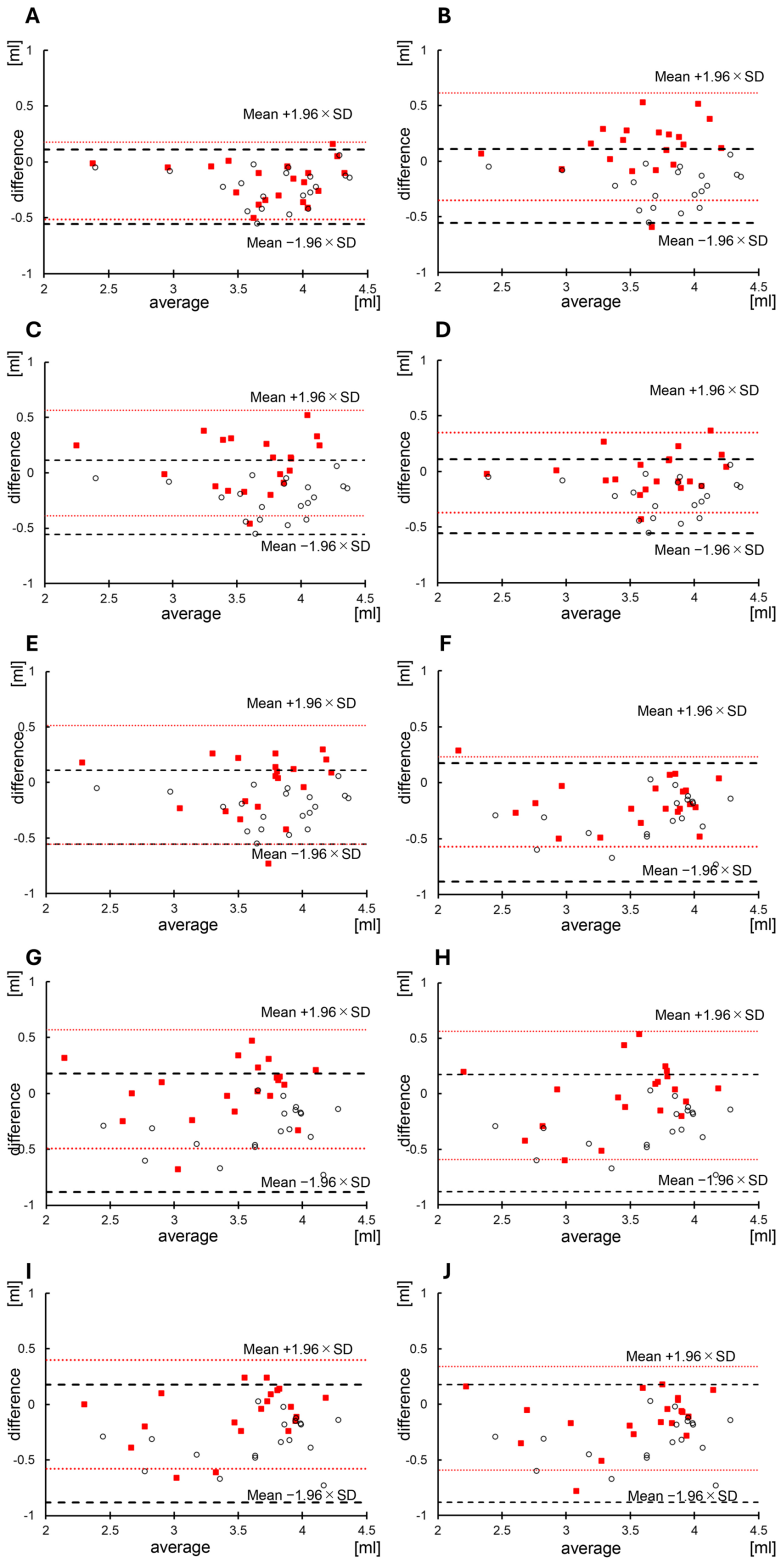
Bland–Altman analysis of the hippocampal volume in input and generated images. The Brandt-Altman analysis results are shown below: **(A)** Left hippocampal volumes between the input and original images and between the generated super-resolution (SR) (λ = e^2^) and original images. **(B)** Left hippocampal volumes between the input and original images and between the generated SR (λ = e^3^) and original images. **(C)** Left hippocampal volumes between the input and original images and between the generated SR (λ = 5 × e^3^) and original images. **(D)** Left hippocampal volumes between the input and original images and between the generated SR (λ = e^4^) and original images. **(E)** Left hippocampal volume between the input and original images and between the generated SR (λ = e^5^) and original images. **(F)** Right hippocampal volume between the input and original images and the generated SR (λ = e^2^) and original images. **(G)** Right hippocampal volume between the input and original images and between the generated SR (λ = e^3^) and original images. **(H)** Right hippocampal volumes between the input and original images and between the generated SR (λ = 5 × e^3^) and original images. **(I)** Right hippocampal volume between the input and original images and between the generated SR (λ = e^4^) and original images. **(J)** Right hippocampal images between the input and original images and the generated SR (λ = e^5^) and original images. The dashed lines and white plots indicate the association between the hippocampal volume analyzed using the input image and the hippocampal volume measured using the original image within the limits of agreement ± 1.96 standard deviations (SD). The dotted lines and red plots represent the association between the hippocampal volume analyzed using the generated SR image (λ = e^5^) and the hippocampal volume measured using the original image within the limits of agreement ± 1.96 SD.

Table 3 shows the left and right hippocampal volumes measured using the BAAD software on the original, input, and generated images. The right and left hippocampal volumes on the original images (mean ± SD) were 3.66 ± 0.47 and 3.45 ± 0.56 mL, respectively. The left and right hippocampal volumes on the input images (64 × 64) were 3.88 ± 0.48 and 3.80 ± 0.48 mL, respectively. The left hippocampal volumes on the generated images were 3.83 ± 0.49 mL at *λ* = e^2^, 3.52 ± 0.42 at λ = e^3^, 3.57 ± 0.46 mL at λ = 5 × e^3^, 3.67 ± 0.45 at λ = e^4^, and 3.68 ± 0.45 at λ = e^5^. There was no significant difference in terms of the hippocampal volume between the original and generated images at *λ* = 5 × e^3^ at *λ* = e^4^ and λ = e^5^ (*p* = 0.10, *p* = 0.76, *p* = 1.00, respectively, Wilcoxon signed-rank test). By contrast, the right hippocampal volumes were 3.62 ± 0.58 mL at *λ* = e^2^, 3.41 ± 0.51 mL at λ = e^3^, 3.46 ± 0.48 mL at λ = 5 × e^3^, 3.54 ± 0.48 mL at λ = e^4^, and 3.58 ± 0.51 mL at *λ* = e^5^. There was no significant difference in terms of the hippocampal volume between the original and generated images at *λ* = e^3^, at *λ* = 5 × e^3^ and λ = e^4^ (*p* = 0.37, *p* = 0.99, *p* = 0.19, respectively). Table 4 and Figure 3 show the results of the Brand-Altman analyses performed separately for the left and right hippocampi. For each hemisphere, comparisons were made between the original and input images, as well as between the original and generated image. For the left hippocampus, the mean bias and 95% limits of agreement [LoA] were −0.22 [−0.56 to 0.11] for the input image. For the generated images, the LoA values at each *λ* were as follows: −0.17 [−0.51 to 0.18] at λ = e^2^, 0.13 [−0.35 to 0.62] at λ = e^3^, 0.091 [−0.39 to 0.57] at *λ* = 5 × e^3^, −0.0090 [−0.37 to 0.35] at λ = e^4^, and −0.021 [−0.56 to 0.51] at λ = e^5^. For the right hippocampus, the mean bias and LoA were −0.35 [−0.88 to 0.18] for the input image. For the generated images, the corresponding values were: −0.17 [−0.57 to 0.23] at λ = e^2^, 0.040 [−0.49 to 0.57] at λ = e^3^, −0.013 [−0.59 to 0.56] at *λ* = 5 × e^3^, −0.090 [−0.58 to 0.40] at λ = e^4^, and −0.12 [−0.59 to 0.34] at *λ* = e^5^.

**Table 3:** Left and right hippocampal volumes.

Image type	Left hippocampal volume (mL) mean ± SD	Original image vs. generated (input) image	Right hippocampal volume (mL) mean ± SD	Original image vs. generated (input) image
Original image (256 × 256)	3.66 ± 0.47		3.45 ± 0.56	
Input image (64 × 64)	3.88 ± 0.48	*P* < 0.05	3.80 ± 0.48	*P* < 0.05
SR image generated at λ = e^2^	3.83 ± 0.49	*P* < 0.05	3.62 ± 0.58	*P* < 0.05
SR image generated at λ = e^3^	3.52 ± 0.42	*P* < 0.05	3.41 ± 0.51	*P* = 0.37
SR image generated at λ = 5 × e^3^	3.57 ± 0.46	*P* = 0.10	3.46 ± 0.48	*P* = 0.99
SR image generated at λ = e^4^	3.67 ± 0.45	*p* = 0.76	3.54 ± 0.48	*P* = 0.19
SR image generated at λ = e^5^	3.68 ± 0.45	*P* = 1.00	3.58 ± 0.51	*p < 0.05*

SD, standard deviation; SR, super resolution.

**Table 4:** Results of the Bland–Altman analysis of the left and right hippocampal volumes.

Image type	Left hippocampal volume (mL)		Right hippocampal volume (mL)	
	Mean bias	Limits of agreement	Mean bias	Limits of agreement
Input image (64 × 64)	−0.22	−0.56 to 0.11	−0.35	−0.88 to 0.18
SR image generated at λ = e^2^	−0.17	−0.51 to 0.18	−0.17	−0.57 to 0.23
SR image generated at λ = e^3^	0.13	−0.35 to 0.62	0.040	−0.49 to 0.57
SR image generated at λ = 5 × e^3^	0.091	−0.39 to 0.57	−0.013	−0.59 to 0.56
SR image generated at λ = e^4^	−0.0090	−0.37 to 0.35	−0.090	−0.58 to 0.40
SR image generated at λ = e^5^	−0.021	−0.56 to 0.51	−0.12	−0.59 to 0.34

SR, super resolution.

## 4 Discussion

### 4.1 Influence of a hyperparameter on hippocampal volume

To reduce MRI acquisition time, the influence of a hyperparameter on hippocampal volume was investigated by modifying the value of *λ* as an adjustment for the Pix2Pix network for super-resolution three-dimensional T1WI reconstruction. In this study, a L1 loss was incorporated into the Pix2Pix model to suppress high-frequency artifacts in super-resolution MRI, with its weight adjusted using the hyperparameter λ. This approach was necessary because using only the adversarial loss leads to the occurrence of high-frequency artifacts, making it essential to include L1 loss as regularization. The hyperparameter *λ* determines the weight of this reconstruction loss (23). High-frequency components in MRI images primarily contribute to contours and boundaries (24), which are considered to have a significant impact on the differentiation between white and gray matter. Therefore, in this study, the initial investigation focused on optimizing the hyperparameter λ. In the objective evaluation, the highest value was obtained at λ = e^3^ (PSNR: 27.91 ± 1.78 dB, MS-SSIM: 0.96 ± 0.0045). By contrast, when super-resolution images were generated at λ = 5 × e^3^, λ = e^4^, the left and right hippocampal volumes were 3.57 ± 0.46 mL and 3.67 ± 0.45 mL, and 3.46 ± 0.48 mL and 3.54 ± 0.48 mL, respectively. There was no significant difference between these values and the hippocampal volumes calculated on the original images (left, *p* = 0.10, *p* = 0.76; right, *p* = 0.99, *p* = 0.19). Mosaic-like generation defects were observed in all or part of the super-resolution images generated using Pix2Pix at *λ* = e^0^ to e^3^. This is because the stability of the training of GANs, including Pix2Pix, decreased based on the values of the hyperparameter input (11, 25). Using the formula for loss function, the GAN was unable to learn properly due to the biased network learning progress because the generator and discriminator were not trained alternately due to excessive weight being placed on the discriminator rather than on the generator. The primary objective of this study was to evaluate whether hippocampal volume accuracy required for clinical BAAD analysis can be maintained in super-resolution images generated by our GAN-based approach. Analysis of hippocampal volume confirmed that measurement accuracy was preserved across a range of *λ* values. However, when the *λ* value was excessively low (e.g., λ = e^2^), mosaic-like noise was observed in some outputs, which may compromise clinical interpretation despite the preservation of volume accuracy. To reduce the risk of such noise while maintaining measurement precision, we recommend setting *λ* to e^4^ when applying the Pix2Pix network to super-resolution processing of medical images. This setting achieves a practical balance between adversarial learning and data fidelity, ensuring both reliable hippocampal volume quantification and clinically interpretable image quality.

### 4.2 Objective evaluation

Zhao et al. reported that the average PSNRs for 4x super-resolution images using MRI was 28.10 db for enhanced super-resolution generative adversarial network (ESRGAN) and 27.41 db for super-resolution generative adversarial network (SRGAN) (26). In the Pix2Pix network used in this study, the PSNR was similar to that of ESRGAN and SRGAN at λ = e^2^ or greater. Therefore, we assume that the performance of 4 × super-resolution three-dimensional T1WI using Pix2Pix is similar to that of other GANs.

In the report of Ledig et al., who first proposed the SRGAN model (12), a hyperparameter equal to *λ* was implemented as e^3^ and validated. In this study, the PSNR and MS-SSIM had the highest values at λ = e^3^, and the objective evaluation results supported the use of the value of *λ* implemented in the GAN-based networks for super-resolution MRI.

### 4.3 Hippocampal volume

Some studies combining VBM and artificial intelligence have used machine learning methods to classify patients with schizophrenia and healthy individuals. Meanwhile, others have utilized VBM to differentiate the white from the gray matter of the brain and identify asymmetry (27). To date, no study has used super-resolution imaging and VBM to evaluate brain volume. The current study calculated hippocampal volumes using low-resolution input images. Results showed that the left and right hippocampal volumes on the low-resolution input images were significantly larger than those on the original image. Moreover, in the Bland–Altman analysis, there were no mean biases in the left and right hemispheres. Based on these findings, low-resolution hippocampal images may underestimate hippocampal atrophy. When the images were generated at *λ* = e^2^, the hippocampal volume measured from the downsampled input images was larger than that calculated from the original images. Additionally, the mean bias and limits of agreement for the input images and those generated at *λ* = e^2^ were as follows: for the input images, the mean bias was −0.22, with limits of agreement ranging from −0.56 to 0.11, and for the images generated at λ = e^2^, the mean bias was −0.17, with limits of agreement ranging from −0.51 to 0.18. This discrepancy is likely due to the reduced contribution of the L1 loss term in the loss function, which prevented the learning process from converging as intended. Consequently, we consider that the super-resolved images generated at λ = e^2^ exhibited hippocampal volumes similar to those measured from the downsampled input images, leading to overestimation. Nevertheless, there was no significant difference in the left and right hippocampal volumes between the image generated at λ = 5 × e^3^ and those at λ = e^4^ and the original image. In addition, the average bias for the left and right hemispheres was close to zero (λ = 5 × e^3^: 0.091 vs. −0.013, λ = e^4^: −0.0090 vs. −0.090). when considering L1 regularization, the intermediate signal intensity is more likely to be applied to the generated image when the edges are unclear (13). Further, this effect becomes more evident as λ increases. Therefore, if the value of λ becomes extremely large and intermediate colors are applied, the contrast between the white and gray matter may become unclear, and the error in volume measurement via VBM analysis can increase.

### 4.4 Prospects for super-resolution technology and clinical applications

High-quality T1-weighted images are essential for the diagnosis of AD achieving this requires extensive k-space encoding and additional T1 imaging, which in turn leads to longer scan times. Techniques such as generalized auto calibrating partially parallel acquisitions (GRAPPA) and, more recently, compressed sensing have been reported to significantly reduce scan times. For example, GRAPPA acceleration has reduced the scan time from 10 min 47 s (with full sampling) to 6 min 17 s, and compressed sensing can further decrease scan times—up to a 10-fold acceleration can reduce the time to as little as 2 min 9 s (28, 29). However, high acceleration rates may lead to trade-offs in image quality and the introduction of artifacts. Reducing the matrix size from 256 to 64 corresponds to reducing the number of phase-encoding steps to 0.25 times the original. In our study, the proposed method yielded hippocampal volumes that did not differ significantly from those of the original images, and the PSNR values were 27.91 ± 1.78 at e^3^, 27.10 ± 1.69 at 5 × e^3^, and 27.65 ± 1.78 at e^4^, indicating minimal degradation in image quality. Theoretically, with all other parameters held constant, this reduction is expected to decrease the scan time to approximately 25% of the original duration, indicating that the proposed approach is potentially beneficial.

Regarding the computational requirements, running the Pix2Pix network does not require specialized servers and can be performed on commercially available PCs, making it accessible for clinical use. Training the model takes approximately 8 h and 30 min, but once trained, generating super-resolution images from 5,120 input images takes 126 s. This short processing time for image generation enables the reduction of MRI scan times while allowing for the creation of high-resolution images without introducing significant delays. This capability supports the feasibility of both reducing scan times and efficiently generating high-quality images, making the approach highly suitable for diagnostic applications.

The down-sampling method used in this study may introduce consistent artifacts in low-resolution scans, which could be misinterpreted by the deep learning algorithm as clues to high-resolution features, potentially leading to reduced performance when applied to real-world data (30). However, the primary objective of this study was not to eliminate artifacts but rather to accurately measure volumes using BAAD software. Our results indicate that the hippocampal volumes derived from the super-resolved images—when appropriate parameter adjustments were made did not significantly differ from those of the original images, suggesting that the impact of consistent artifacts in low-resolution scans is minimal.

In AD diagnosis, hippocampal volume is a well-established biomarker; however, other critical indicators also play a significant role. For instance, the amygdala undergoes structural changes at various stages of disease progression (31). Recent studies have increasingly focused on leveraging artificial intelligence for staging AD (32). The integration of structural MRI and resting-state functional MRI (rs-fMRI) enables more precise diagnostic analysis and classification. By combining hippocampal subfields, amygdala volume, and brain network features with multiple rs-fMRI metrics, the accuracy of AD diagnosis can be significantly improved (33). Moreover, Wang, et al., a network that effectively integrates a pre-training module (Transformer) with a self-training module (convolutional neural network) in an interactive manner demonstrated superior performance compared to using convolutional or attention mechanisms alone. This approach balances computational efficiency while preserving both local and global features. As deep learning algorithms evolve through the integration of new data, there is significant potential for further advancements (34). While our current research focuses on GANs, we plan to explore various learning models in future studies. As deep learning evolves, exploring a variety of architectures other than GANs could lead to more accurate and efficient diagnostic tools.

In summary, *λ* = e^3^ had the highest value in the objective evaluation. However, the learning of some images was unstable. By setting at λ = e^4^, the images were stabilized, and hippocampal volumes that were close to those on the original images that had high objective evaluation scores were calculated. Therefore, λ = e^4^ is the optimal value for super-resolution using the Pix2Pix network. The strength of this study is that it used super-resolution images to measure the hippocampal volume via VBM analysis. In addition, it directly compared the hippocampal volume measured using super-resolution images with that measured using the original image. In addition, as a clinical application of this research, the reduction in acquisition time for 3D T1WI MRI is beneficial for not only patients with AD who have difficulty undergoing long MRI scans but also others such as those with claustrophobia and elderly individuals. In addition, this technology can achieve an image with a super resolution without the need for any special hardware or software. Thus, it can also be applied to images that have been taken in the past.

The current study had several limitations. First, the Pix2Pix network was trained not on actual low-resolution MRI scans but on pseudo-low-resolution images derived from a high-resolution dataset. Obtaining paired low-resolution and high-resolution MRI scans from the same patient, particularly those with AD, is extremely difficult. While this simulation-based approach may limit the real-world applicability, it is commonly used in cases where real-world paired data are lacking (35). Future work will focus on acquiring paired low-resolution and high-resolution MRI scans from actual MRI images of healthy volunteers to further validate the model. Second, the dataset selected from OASIS-3 only included 3-T MRI images and primarily consisted of AD and healthy control subjects. In this study, using only 3-Tesla data may not capture potential differences in image quality or tissue contrast that can occur with other magnetic field strengths, thereby restricting the generalizability of our findings. Third, the study focused on AD patients, leaving uncertainty about whether the proposed super-resolution technique is applicable to other populations, such as patients with non-AD dementias or different age groups. To address these limitations, future studies will assess its generalizability using diverse datasets covering various neurodegenerative diseases and demographic variations. Additionally, the model’s performance will be evaluated on MRI data acquired at different field strengths, and the dataset will be expanded using publicly available resources such as ADNI (https://adni.loni.usc.edu/) and Kaggle (https://www.kaggle.com/).

## 5 Conclusion

Using low-resolution images is one of the approaches for reducing MRI acquisition time. In this study, when hippocampal volume was measured directly from low-resolution images, it was more likely to be overestimated, and brain atrophy was underestimated. Therefore, an objective evaluation was performed, and changes in hippocampal volume were investigated by adjusting *λ*, the main hyperparameter of the super-resolution Pix2Pix network. The optimal value was at λ = e^4^, which resulted in a high objective evaluation. BAAD-based hippocampal volume measurements from super-resolution images showed no significant differences from the original images, effectively reducing measurement errors. These results suggest that the proposed method has the potential to reduce MRI acquisition time and patient burden by enabling accurate hippocampal volume measurement from super-resolution images. However, given the limitations of training on synthetic data and the small dataset size, further validation using real-world clinical data is necessary. Additionally, future studies will explore alternative optimization strategies beyond λ selection to improve image quality and robustness.

## Data Availability

The datasets presented in this study can be found in online repositories. The names of the repository/repositories and accession number(s) can be found at: OASIS-3 (https://sites.wustl.edu/oasisbrains/).
